# RAS-pathway mutations are common in patients with ruxolitinib refractory/intolerant myelofibrosis: molecular analysis of the PAC203 cohort

**DOI:** 10.1038/s41375-023-02027-3

**Published:** 2023-10-20

**Authors:** J. M. O’Sullivan, J. Taylor, A. Gerds, S. Buckley, C. N. Harrison, S. Oh, A. F. List, K. Howard, H. Dreau, A. Hamblin, A. J. Mead

**Affiliations:** 1 NIHR Biomedical Research Centre and MRC Molecular Haematology Unit, Weatherall Institute of Molecular Medicine, University of Oxford, Oxford, UK; 2 Department of Clinical Haematology, Guy’s and St Thomas NHS Foundation Trust, London, UK; 3 Astex Pharmaceuticals, Inc., Pleasanton, CA USA; 4 Leukemia Program, Department of Hematology and Medical Oncology, Taussig Cancer Institute, Cleveland Clinic Taussig Cancer Institute, Cleveland, OH USA; 5 CTI BioPharma Corp., Seattle, WA USA; 6 Washington University School of Medicine, Saint Louis, MO USA; 7 Precision BioSciences, Inc., Durham, NC USA; 8 Department of Haematology, Oxford University Hospitals NHS Foundation Trust, Oxford, UK

**Keywords:** Cancer genomics, Myeloproliferative disease, Targeted therapies

## To the Editor:

The treatment of myelofibrosis (MF), a myeloproliferative neoplasm (MPN) driven by JAK-STAT pathway activating mutations, evolved with the advent of JAK inhibitors. The first-in-class agent, ruxolitinib (RUX), a JAK1/2 inhibitor, is now standard for treatment of splenomegaly and MF-associated symptoms [1]. However, MF is a biologically and clinically heterogeneous disease with certain difficult to treat patient subgroups. In particular, disease- or treatment-associated thrombocytopenia is associated with adverse outcomes [2, 3] and often requires RUX dose reductions or interruptions which may limit treatment efficacy. Thrombocytopenic patients who discontinue RUX have a median survival of less than 1 year [3].

Pacritinib (PAC), a JAK2/IRAK1/ACVR1 inhibitor that spares JAK1, has shown clinical benefit in thrombocytopenic MF in the PERSIST-1 and -2 trials [4, 5]. PAC203 was a randomized dose finding study of PAC in primary or secondary MF patients who were refractory or intolerant to RUX (RUX-ref/int), including patients with moderate and severe thrombocytopenia. Patients were randomized 1:1:1 (PAC 100 mg once daily [QD], 100 mg twice daily [BID] or 200 mg BID) stratified by baseline platelet count. This study established PAC 200 mg BID as the optimal efficacious and safe dose [6] and PAC is now FDA approved for the treatment of patients with MF who have thrombocytopenia.

Previous studies established the adverse prognostic implications of certain somatic gene mutations in MF; specifically mutations in epigenetic (*ASXL1*, *EZH2*) [7], splicing factor (*SRSF2*, *U2AF1*) [7] and *IDH1*/*IDH2* genes [7] are associated with disease progression and shortened survival. Reduced likelihood of RUX response has been associated with ≥3 mutations [8] but not with mutation type [9, 10]. Shorter time to RUX failure has been reported in those with *ASXL1*/*EZH2* mutations [9] and reduced time to RUX discontinuation in patients with ≥3 mutations [8]. Specific cytokine signatures have been correlated with RUX resistance [11] suggesting possible biologically relevant pathways (e.g. NFκB) mediating resistance. The mutation profiles of RUX-ref/int thrombocytopenic MF patients have not been well delineated. This represents a group with a major unmet need for effective management strategies, and a better understanding of their mutation profiles will assist the application of precision medicine in this challenging group.

We therefore performed mutational analysis on a subgroup the PAC203 cohort (110 patients at baseline and 42 patients at 24 weeks follow-up using a 32-gene TruSeq Custom Amplicon Panel (see Supplementary Methods). Furthermore, we interrogated cytokine profiles to understand the relationship between inflammatory signatures and clinico-genomic profiles in this cohort.

Characteristics of this group was representative of the overall PAC203 cohort [6]. Median follow-up time was 213 (95% confidence interval [CI]: 189–236) days. The median age was 68 (37–87) years, the median duration of prior exposure to RUX was 1.59 years (range 0–11 years) with 72.7% reporting prior exposure to non-RUX therapies (range 1–5 lines). Primary myelofibrosis (PMF) was the most prevalent disease category (56.4%, 62/110), followed by post-polycythemia vera MF (PPV-MF) in 29.1% (32/110) and post-essential thrombocythemia MF (PET-MF) in 14.5% (16/110). Thrombocytopenia was common: median baseline platelet count was 63 ×10^9^/L, with 38.2% (42/110) <50 ×10^9^/L. Baseline hemoglobin was <10 g/dL in 64.5% (71/110) of the cohort.

**Fig. 1:**
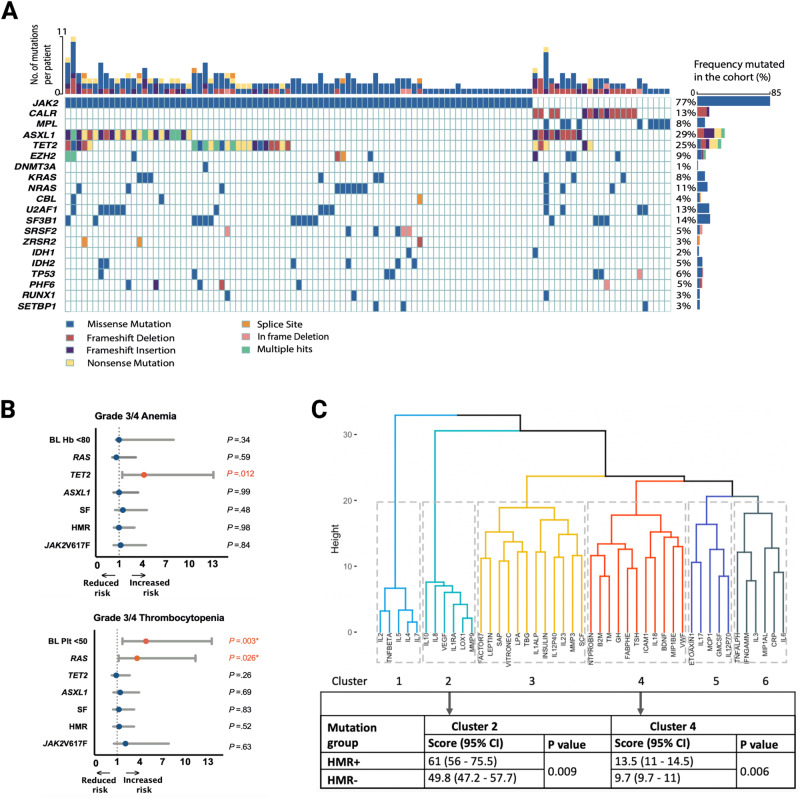
The mutation and cytokine profiles in the PAC203 cohort. **A** Waterfall plot of mutation distribution in the PAC203 cohort. **B** Forest plot illustrating the results of logistic regression analyses of mutation statuses and baseline hemoglobin count associated with the likelihood of grade 3/4 anemia; *TET2*-mutated patients were more likely to experience grade 3/4 anemia independent of baseline hemoglobin level; odds ratio (OR) 4.5, 95% CI 1.4–13.9, *P* = 0.009 (upper panel). Forest plot illustrating the results of logistic regression analyses of mutation statuses and baseline platelet count associated with the likelihood of grade 3/4 thrombocytopenia; *KRAS/NRAS*-mutated patients were more likely to experience grade 3/4 anemia after adjustment for baseline platelet level; OR 3.65, 95% CI 1.2–11.3, *P* = 0.026. (lower panel). Univariate logistic regression was performed for each variable. Significant *P* values (<0.05) highlighted in red and OR denoted by (*) were adjusted. JAK2V617F JAK2 V617F-mutated; HMR high molecular risk mutation [*IDH1/2, SRSF2, ASXL1, EZH2, U2AF1*Q157]; SF splicing factor mutation [*SF3B1*, *U2AF1*, *SRSF2*, *ZRSR2*], *ASXL1* *ASXL1*-mutated; *TET2* *TET2*-mutated, *RAS* *KRAS/NRAS*-mutated, BL Plt <50 baseline platelet level <50 × 10^9^/L. **C** Cluster dendrogram of cytokine levels in ruxolitinib refractory / intolerant study cohort with cluster scores 2 and 4 highlighted in the table for high molecular risk positive (HMR+) and negative (HMR−) patients.

MPN driver mutation frequency was as expected for MF [10, 12]; *JAK2*V617F mutation was present in 77.3% (85/110), *CALR*-mutation in 12.7% (14/110; type 1: *n* = 11, type 2: *n* = 3), *MPL*-mutation in 8.2%, and “triple-negative” in 1.8% of cases, Fig. 1A. *JAK2*V617F variant allele frequency (VAF) was ≥50% in 68.2% (58/85) with VAF < 20% present in just 5.9% (*n* = 5) of patients. Non-MPN driver mutations (NDM) were present in 76.4% (*n* = 84) with ≥3 NDMs in 20.9% (23/110) of patients. Analogous to previous reports, the most prevalent NDMs were in *ASXL1* and *TET2* genes (in 29.1%, *n* = 32, and 26.4%, *n* = 29, of patients respectively) (Fig. 1A, Supplementary Table S1). Splicing factor (SF) gene mutations were mutually exclusive and detected in 34.5% (*n* = 38/110) of patients, which included *SF3B1* [13.6%, *n* = 15], *U2AF1* [12.7%, *n* = 14], *SRSF2* [5.5%, *n* = 6], *ZRSR2* [2.7%, *n* = 3]. Patients with SF mutations were more often categorized as PMF (76.3%) rather than PET-MF (15.8%) or PPV-MF (7.9%), *P* = 0.001 (Supplementary Table S2A). SF-mutated patients had lower baseline hemoglobin level (Hb <8 g/dL in 39.5% as compared with 16.9% in SF-wild type [WT], *P* = 0.009) and were more likely to be red cell transfusion dependent at trial entry (RCC-D) as compared with SF-WT patients (42.1% vs 22.2% respectively, *P* = 0.012; Supplementary Table S2A). *SF3B1*-mutated patients had higher trial entry platelet counts (platelet count >100 × 10^9^/L) in 66.7% vs. 28.7% in *SF3B1*-WT patients, *P* = 0.004).

High molecular risk mutations (HMR; *IDH1/2, SRSF2, ASXL1, EZH2, U2AF1*Q157) [7] were present in 43.6% (48/110) and ≥2 HMR mutations were present in 15.4%, a prevalence similar to other high-risk enriched MF cohorts [12, 13]. No clinical parameters were associated with a HMR mutation (Supplementary Table S2B). Strikingly, RAS-pathway mutations, *KRAS/NRAS/CBL* (*RAS/CBL*-MT), were found at a higher frequency than previously described in MF cohorts [13, 14] in 20.9% of patients (*n* = 23; *RAS* *n* = 20, *CBL* *n* = 3; Fig. 1A, Table 1). These mutations were sub-clonal in the majority with a median VAF of 10% (range 1.9–95%). *RAS* mutations occurred in known mutation hotspots; the most prevalent was in codon G12 (*n* = 12/20) [13]. *RAS*/*CBL*-MT patients had a significantly higher frequency of NDMs (≥3 in 56.5% vs. 9.2% for *RAS*/*CBL*-WT patients, *P* = 0.0001, Table 1) and a co-mutated HMR mutation (65.2% vs 37.9% for *RAS*/*CBL*-WT patients, *P* = 0.02, Table 1). *KRAS/NRAS/CBL* and *TP53* (*n* = 7 patients) mutations were mutually exclusive in this cohort.

**Table 1:** *RAS/CBL*-mutated patient baseline clinical and mutation characteristics.

	*RAS/CBL-*mutated, *N* = 23 *n* (%)	*RAS/CBL-*WT, *N* = 85 *n* (%)	*P* value
Age in years, median (range)	69 (56–85)	68 (37–87)	0.42
Male gender, *n* (%)	14 (60.9)	50 (57.5)	0.77
MF diagnosis, *n* (%)			
Primary MF	13 (56.5)	49 (56.3)	
PPV MF	8 (34.8)	24 (27.8)	
PET MF	2 (8.7)	14 (16.1)	0.6
Prior ruxolitinib, *n* (%)			
Failure	16 (69.6)	64 (73.6)	0.64
Intolerance	18 (78.2)	59 (67.8)	0.33
Prior ruxolitinib duration in months, median (range)	29 (1.7–131.4)	16.6 (1.7–119)	0.38
Platelet count × 10^9^/L, median (range)	59 (14–402)	63.5 (13–910)	0.47
Platelet count <50, ×10^9^/L, *n* (%)	10 (43.5)	32 (36.8)	0.58
Hemoglobin <10 g/dL, *n* (%)	19 (82.6)	52 (59.8)	0.13
RBC transfusion dependent, *n* (%)	10 (43.5)	22 (25.2)	0.25
Platelet transfusion dependent *n* (%)	4 (17.4)	5 (5.7)	0.07
Peripheral blasts, median (range)	2 (1–5)	2 (0–17)	0.65
White blood cells, ×10^9^/L, median (range)	6.6 (1.2–107.7)	6.8 (1.1–103.4)	0.85
Spleen Volume (cm^3^) by MRI/CT, median (range)	2589 (458–5520)	2240 (262–4994)	0.22
Driver mutation status			
*JAK2*V617F	18 (78.2)	67 (77)	
*CALR*	2 (8.7)	12 (13.8)	
*MPL*	2 (8.7)	7 (8)	
Triple negative	1 (4.4)	1 (1.2)	0.7
*JAK2* V617F allele burden ≥50%	12 (66.7)	46 (68.7)	1
HMR mutation	15 (65.2)	33 (37.9)	0.02
NDM ≥3	13 (56.5)	8 (9.2)	0.0001

*WT* wild-type, *MF* myelofibrosis, *PPV* post polycythemia, *PET* post essential thrombocythemia, *RBC* red blood cell, *HMR* high molecular risk, *NDM* non-myeloproliferative neoplasm driver mutation.

In patients with both molecular and 24-week clinical data, there were no significant correlations between driver or NDM mutation status (including specific analyses relating to HMR and *RAS*/*CBL*-MT status) and SVR or TSS response, although numbers of events for analysis were low. Grade 3/4 anemia occurred more often during the study period in *TET2-*mutated patients (odds ratio [OR] 4.2, 95% CI 1.4–13, *P* = 0.012), Fig. 1B. Grade 3/4 thrombocytopenia occurred more frequently in *RAS*/*CBL*-MT patients (OR 3.64, 95% CI 1.2–11.3, *P* = 0.026, Fig. 1B), including after adjusting for baseline platelet strata (< vs. ≥50 ×10^9^/L). The presence of ≥3 NDMs was associated with an increased risk of infections (OR 7.59, 95% CI 2.45–23.4, *P* = 0.0001).

Follow-up molecular analysis at week 24 was performed in 38.2% (*n* = 42/110). No significant driver or NDM molecular responses (≥50% reduction in VAF) were detected. At least one new NDM was acquired in 7.1% (3/42) including *CBL* [2], *TET2* [1], *TP53* [1], *U2AF1* Q157 [1]. No associations were observed between follow-up mutation analyses and outcomes.

Unsupervised clustering identified 6 cytokine clusters at baseline, Fig. 1C. Elevated cluster 2 (*P* = 0.009) and 4 (*P* = 0.006) scores were associated with HMR mutations. Higher cluster 2 scores were also associated with driver mutation VAF ≥50%. The pro-inflammatory cytokines in cluster 2 linked to HMR mutations (HMR+) represented a cluster regulated by the NFκB pathway. The presence of a HMR mutation was particularly associated with significantly higher IL-8 levels (40.5 pg/ml) as compared with absence of an HMR mutation (24.5 pg/ml), *P* < 0.0001. Elevated tumor necrosis factor-alpha (TNF-α) was also associated with HMR mutations; TNF-α was 61 pg/ml in HMR+ vs. 48.5 pg/ml for HMR−, *P* = 0.009. Although RAS-pathway mutations were not associated with specific cluster scores, these patients did have higher levels of the NFκB-associated cytokine IL12P40 (1.1 ng/ml) as compared with *RAS/CBL*-WT patients (0.6 ng/ml), *P* = 0.001. There was no association between cytokine cluster scores and exposure to RUX.

We report the mutation landscape in RUX-ref/int cytopenic MF, showing enrichment for HMR mutations and, in particular, a higher frequency of *RAS*-pathway mutations (20.9%) than previously reported in MF cohorts (to date at a frequency of 6–8.1%) [13, 14]. *RAS* and HMR mutation co-occurrence has previously been described, which we also observed [14]. *RAS*-pathway mutations often showed low allele burden and correlated with the presence of multiple NDMs, consistent with presence of *RAS*-pathway mutations in patients undergoing genetic evolution. Although mutation data was not available prior to RUX treatment in this cohort, recent single cell genetic analyses in myelofibrosis show *RAS*-pathway mutations were one of most common emergent mutations after exposure to RUX [15]. Activating mutations of the *RAS*-pathway have also been reported to correlate with reduced likelihood of spleen and symptom responses in patients with myelofibrosis treated with dual JAK1/2 inhibitors [13]. *RAS*-pathway mutations in MF have also been associated with shorter survival and progression to leukemia [14]. The PAC203 cohort therefore represents a genetically high risk group of patients.

Importantly, we report for the first time a relationship between HMR and *RAS* mutations and a pro-inflammatory cytokine signature. This signature mirrors a previously described RUX resistant cytokine profile [11] involving NFκB signaling. A potential underlying mechanism may be that the inflammatory microenvironment creates a selective pressure promoting the evolution of subclones carrying HMR and RAS-pathway mutations. We speculate that this combination of cell-intrinsic genetic properties of the clone, and cell-extrinsic inflammatory microenvironment might collectively confer JAKi resistance. Therapeutic strategies, including dual blockade of JAK2 and NFκB, may prove beneficial for treatment of MF. As a JAK2/IRAK1 inhibitor, PAC targets both pathways, as IRAK1 is upstream of NFκB signaling suggesting a potential role in those with HMR and *RAS*-pathway mutations. Although no specific TSS or SVR responses were observed in these patients on PAC203, the numbers of patients available for analysis was low, and the follow-up period may not have been sufficient to capture responses in this subgroup. Other strategies, including combinations targeting JAK and MEK/ERK pathways together with inflammatory pathways, for example through bromodomain inhibition, could be an effective strategy to mitigate clonal evolution in high-risk patients.

In summary, the PAC203 cohort encompasses a molecularly high-risk group, with a high incidence of HMR and *RAS* pathway mutations that may be associated with JAK1/2 inhibitor resistance. Our findings will help inform the application of precision medicine for this group of patients with a major unmet need for new therapeutic strategies.

### Supplementary information

Supplemental Methods

Supplemental Table 1

Supplemental Table 2A

Supplemental Table 2B
