# Supplementary material for: RAS-pathway mutations are common in patients with ruxolitinib refractory/intolerant myelofibrosis: molecular analysis of the PAC203 cohort
Source: Leukemia. 2023 Oct 20;37(12):2497–501. doi: 10.1038/s41375-023-02027-3 (PMC10681886; doi:10.1038/s41375-023-02027-3)
Supplement: Supplementary file 1 — Supplemental Methods [file 41375_2023_2027_MOESM1_ESM.docx]

**Supplemental Methods**

Genomic DNA (gDNA) was isolated from peripheral blood at baseline in 110 patients (of total 164 recruited and 161 treated) and at 24 weeks in 42 patients. Mutational profiles of driver and non-MPN driver (NDM) mutations were analyzed using an International Organization for Standardization Standardization (ISO 15189:2012) accredited Illumina TruSeq Custom Amplicon Panel which included 32 gene mutation hotspots & exons commonly mutated in myeloid neoplasms (~56,000 bp, 341 amplicons); *ASXL1, ATRX, DNMT3A, EZH2, TET2, CEBPA, ETV6, NPM1, PHF6, RUNX1, SETBP1, SF3B1, SRSF2, TP53, U2AF1, WT1, ZRSR2, CBL, CBLB, CBLC, CSF3R, FLT3, HRAS, JAK2, KIT, KRAS, MPL, NRAS, PDGFRA, PTEN, IDH1, IDH2.^1^*

Paired-end indexed libraries were sequenced on the Illumina platform. FASTQ files were aligned to the reference genome (GRCh37/hg19). The minimum depth was ≥100 reads per base and minimum accepted coverage was achievement of this depth in ≥95% of targeted bases. Acceptable coverage was achieved in 99.1% (109/110) of patients. A custom designed in-house algorithm was used to evaluate variants. Pathogenic variants were reported at a variant allele frequency of ≥1% and pathogenic significance of each variant was determined using genome aggregation database (gnomAD v2.1.1),^2^ the Single Nucleotide Polymorphism database (dbSNP), the Catalogue Of Somatic Mutations In Cancer (COSMIC) databases and published literature. Variants considered pathogenic included those reported as somatic in COSMIC and not found in germline databases, those present at the same genomic location of a somatic mutation reported in COSMIC, those not reported in dbSNP databases with a variant allele frequency (VAF) <40-45% but predicted to result in a truncated protein. Single-nucleotide variations (SNVs) were excluded if the population frequency was >1% or if the population frequency of <1% but with ethnicity bias and a variant allele frequency (VAF) close to 50%. Cytokine data were available in 108 patients.  Using the Myriad RBM platform, a microsphere-based immuno-multiplexing technology, 47 cytokines were assessed; B2M, BDNF, CRP, ETOAXIN1, FABPHE, FACTOR7, GH, GMCSF, ICAM1, IFN- γ, IL-10, IL-12P40, IL-12P70, IL-15, IL-17, IL-18, IL-1ALP, IL-1-β, IL-1RA, IL-2, IL-23, IL-3, IL-4, IL-5, IL-6, IL-7, IL-8, insulin, leptin, LOX1, LPA, MCP1, MIP1AL, MIP1BE, MMP3, MMP9, NTPROBN, SAP, SCF, TBG, TM, TNF-α, TNF-β, TSH, VEGF, vitronectin and VWF.

Driver and NDM were analyzed as individual mutations and mutation groups (e.g. epigenetic, splicing factor). Univariate and multivariate logistic regression was performed to examine associations between mutation status and treatment response and adverse events. Responses at 24 weeks were defined by reduction in spleen volume of ≥35% as assessed by MRI or CT and reduction in total symptom score (using MPN-SAF TSS 2.0^3^) of≥50%. Adverse events were included in this analysis if grade 3 or 4 (classified by the National Cancer Institute Common Terminology Criteria for Adverse Events v4.03. The reverse Kaplan-Meier method was used to determine the median follow-up time with the event of interest reversed. Survival outcome analyses were not performed due to short follow-up period. Individual plasma cytokine levels were correlated with specific somatic gene mutations and clinical demographic data followed by an unsupervised approach which applied hierarchical agglomerative clustering (using *hclust* function in stats R package, with “ward.D2” method) to identify related sets of cytokines. A cluster score was derived based on the median overall cytokine concentration within a cluster for each patient. Scores were then correlated clinico-genomic data was analyzed. Statistical analyses were performed using SPSS version 26 and R statistical software package version 3.6.0 with RStudio version 1.1.463.

1. Hamblin A, Burns A, Tham C, et al. Development and Evaluation of the Clinical Utility of a Next Generation Sequencing (NGS) Tool for Myeloid Disorders. *Blood.* 2014;124(21):2373-2373.

2. Karczewski KJ, Francioli LC, Tiao G, et al. The mutational constraint spectrum quantified from variation in 141,456 humans. *Nature.* 2020;581(7809):434-443.

3. Emanuel RM, Dueck AC, Geyer HL, et al. Myeloproliferative neoplasm (MPN) symptom assessment form total symptom score: prospective international assessment of an abbreviated symptom burden scoring system among patients with MPNs. *J Clin Oncol.* 2012;30(33):4098-4103.
