# Supplementary material for: RAS-pathway mutations are common in patients with ruxolitinib refractory/intolerant myelofibrosis: molecular analysis of the PAC203 cohort
Source: Leukemia. 2023 Oct 20;37(12):2497–501. doi: 10.1038/s41375-023-02027-3 (PMC10681886; doi:10.1038/s41375-023-02027-3)
Supplement: Supplementary file 2 — Supplemental Table 1 [file 41375_2023_2027_MOESM2_ESM.docx]

| **Tumor_Sample_Barcode** | **Variant_Classification** | **Hugo_Symbol** | **Chr** | **HGVSp** |
| --- | --- | --- | --- | --- |
| 44001-3092 | Nonsense_Mutation | TET2 | 4 | NP_001120680.1:p.Gln255Ter |
| 44001-3092 | Missense_mutation | JAK2 | 9 | NP_004963.1:p.Val617Phe |
| 44001-3089 | Nonsense_Mutation | TET2 | 4 | NP_001120680.1:p.Gln373Ter |
| 44001-3089 | Missense_mutation | JAK2 | 9 | NP_004963.1:p.Val617Phe |
| 44001-3089 | Missense_mutation | RUNX1 | 21 | NP_001745.2:p.Arg162Gly |
| 44001-3089 | In_Frame_Del | SRSF2 | 17 | NP_001182356.1:p.Pro95_Arg102del |
| 36003-3066 | Missense_mutation | JAK2 | 9 | NP_004963.1:p.Val617Phe |
| 36003-3126 | Missense_mutation | SF3B1 | 2 | NP_036565.2:p.Lys666Thr |
| 36003-3126 | Missense_mutation | EZH2 | 7 | NP_004447.2:p.Tyr741Cys |
| 36003-3126 | Missense_mutation | EZH2 | 7 | NP_004447.2:p.Cys554Arg |
| 36003-3126 | Missense_mutation | JAK2 | 9 | NP_004963.1:p.Val617Phe |
| 36003-3126 | Frame_Shift_Ins | ASXL1 | 20 | NP_056153.2:p.Glu790Ter |
| 36003-3126 | Frame_Shift_Del | TET2 | 4 | NP_001120680.1:p.Lys203ArgfsTer4 |
| 36003-3126 | Nonsense_Mutation | EZH2 | 7 | NP_004447.2:p.Ser644Ter |
| 36003-3100 | Missense_mutation | JAK2 | 9 | NP_004963.1:p.Val617Phe |
| 36003-3100 | Frame_Shift_Ins | ASXL1 | 20 | NP_056153.2:p.Gly646TrpfsTer12 |
| 36003-3080 | Missense_mutation | EZH2 | 7 | NP_004447.2:p.Ala682Glu |
| 36003-3080 | Frame_Shift_Del | CALR | 19 | NP_004334.1:p.Leu367ThrfsTer46 |
| 36003-3108 | Missense_mutation | JAK2 | 9 | NP_004963.1:p.Val617Phe |
| 36003-3108 | Nonsense_Mutation | TET2 | 4 | NP_001120680.1:p.Arg1404Ter |
| 36003-3108 | Frame_Shift_Del | ASXL1 | 20 | NP_056153.2:p.Glu635ArgfsTer15 |
| 36003-3082 | Missense_mutation | NRAS | 1 | NP_002515.1:p.Gly12Asp |
| 36003-3082 | Splice_site | EZH2 | 7 |  |
| 36003-3082 | Missense_mutation | JAK2 | 9 | NP_004963.1:p.Val617Phe |
| 36003-3079 | Missense_mutation | JAK2 | 9 | NP_004963.1:p.Val617Phe |
| 36003-3079 | Frame_Shift_Del | ZRSR2 | X | NP_005080.1:p.Lys26AsnfsTer12 |
| 36003-3079 | Splice_site | CBL | 11 |  |
| 34001-3104 | Missense_mutation | JAK2 | 9 | NP_004963.1:p.Val617Phe |
| 34001-3104 | Missense_mutation | IDH2 | 15 | NP_002159.2:p.Arg140Gln |
| 34001-3104 | Missense_mutation | SRSF2 | 17 | NP_001182356.1:p.Pro95Leu |
| 34001-3104 | Missense_mutation | NRAS | 1 | NP_002515.1:p.Gly12Ser |
| 34001-3129 | Missense_mutation | JAK2 | 9 | NP_004963.1:p.Val617Phe |
| 44007-3107 | Missense_mutation | JAK2 | 9 | NP_004963.1:p.Val617Phe |
| 44006-3132 | Frame_Shift_Del | TET2 | 4 | NP_001120680.1:p.Glu263ArgfsTer30 |
| 44006-3132 | Missense_mutation | JAK2 | 9 | NP_004963.1:p.Val617Phe |
| 44005-3143 | Missense_mutation | SF3B1 | 2 | NP_036565.2:p.Lys666Asn |
| 44005-3143 | Missense_mutation | JAK2 | 9 | NP_004963.1:p.Val617Phe |
| 10060-3084 | Frame_Shift_Ins | TET2 | 4 | NP_001120680.1:p.Arg1440ThrfsTer38 |
| 10060-3084 | Missense_mutation | JAK2 | 9 | NP_004963.1:p.Val617Phe |
| 10035-3035 | Missense_mutation | JAK2 | 9 | NP_004963.1:p.Val617Phe |
| 10060-3102 | Missense_mutation | JAK2 | 9 | NP_004963.1:p.Val617Phe |
| 10060-3102 | Missense_mutation | U2AF1 | 21 | NP_001020374.1:p.Ser34Phe |
| 10060-3098 | Missense_mutation | MPL | 1 | NP_005364.1:p.Ser505Asn |
| 10060-3098 | In_Frame_Del | TP53 | 17 | NP_000537.3:p.His179del |
| 10060-3098 | Missense_mutation | U2AF1 | 21 | NP_001020374.1:p.Gln157Pro |
| 10060-3099 | Missense_mutation | NRAS | 1 | NP_002515.1:p.Gly12Asp |
| 10060-3099 | Missense_mutation | JAK2 | 9 | NP_004963.1:p.Val617Phe |
| 10048-3116 | Missense_mutation | JAK2 | 9 | NP_004963.1:p.Val617Phe |
| 10048-3116 | Missense_mutation | KRAS | 12 | NP_203524.1:p.Gln61Arg |
| 10048-3116 | Missense_mutation | SRSF2 | 17 | NP_001182356.1:p.Pro95His |
| 10048-3116 | Missense_mutation | SETBP1 | 18 | NP_056374.2:p.Gly870Ser |
| 10015-3078 | Missense_mutation | IDH1 | 2 | NP_005887.2:p.Arg132His |
| 10015-3078 | Missense_mutation | JAK2 | 9 | NP_004963.1:p.Val617Phe |
| 10015-3078 | In_Frame_Del | SRSF2 | 17 | NP_001182356.1:p.Pro95_Arg102del |
| 10025-3052 | Missense_mutation | U2AF1 | 21 | NP_001020374.1:p.Gln157Pro |
| 10025-3052 | Missense_mutation | U2AF1 | 21 | NP_001020374.1:p.Gln157His |
| 10025-3052 | Missense_mutation | JAK2 | 9 | NP_004963.1:p.Val617Phe |
| 10025-3052 | Missense_mutation | TP53 | 17 | NP_000537.3:p.Phe270Ser |
| 10035-3070 | Missense_mutation | JAK2 | 9 | NP_004963.1:p.Val617Phe |
| 10035-3070 | v | CBL | 11 | NP_005179.2:p.Leu380Pro |
| 10035-3070 | Nonsense_mutation | ASXL1 | 20 | NP_056153.2:p.Gln733Ter |
| 10035-3063 | Missense_mutation | TET2 | 4 | NP_001120680.1:p.Ser1284Ala |
| 10035-3063 | Missense_mutation | TET2 | 4 | NP_001120680.1:p.Phe1285Ser |
| 10035-3063 | Missense_mutation | EZH2 | 7 | NP_004447.2:p.Ala682Val |
| 10035-3063 | Frame_Shift_Del | EZH2 | 7 | NP_004447.2:p.Ile459LeufsTer4 |
| 10035-3063 | Missense_mutation | JAK2 | 9 | NP_004963.1:p.Val617Phe |
| 10035-3063 | Missense_mutation | CBL | 11 | NP_005179.2:p.Cys396Tyr |
| 10035-3063 | Frame_Shift_Del | ASXL1 | 20 | NP_056153.2:p.Ala640GlyfsTer14 |
| 10035-3063 | Frame_Shift_Ins | ASXL1 | 20 | NP_056153.2:p.Gly646TrpfsTer12 |
| 10035-3063 | Frame_Shift_Del | ASXL1 | 20 | NP_056153.2:p.Leu731TyrfsTer13 |
| 10035-3063 | Nonsense_mutation | ASXL1 | 20 | NP_056153.2:p.Leu775Ter |
| 10035-3063 | Missense_mutation | U2AF1 | 21 | NP_001020374.1:p.Gln157Pro |
| 10065-3059 | Frame_Shift_Del | TET2 | 4 | NP_001120680.1:p.Asn595LeufsTer41 |
| 10065-3059 | Missense_mutation | JAK2 | 9 | NP_004963.1:p.Val617Phe |
| 10066-3110 | Missense_mutation | MPL | 1 | NP_005364.1:p.Trp515Leu |
| 10062-3061 | Frame_Shift_Del | EZH2 | 7 | NP_004447.2:p.Pro257LeufsTer8 |
| 10062-3061 | Missense_mutation | JAK2 | 9 | NP_004963.1:p.Val617Phe |
| 10062-3061 | Missense_mutation | NRAS | 1 | NP_002515.1:p.Gly12Asp |
| 10062-3076 | Missense_mutation | NRAS | 1 | NP_002515.1:p.Gly12Asp |
| 10062-3076 | Missense_mutation | JAK2 | 9 | NP_004963.1:p.Val617Phe |
| 10062-3076 | Nonsense_mutation | ASXL1 | 20 | NP_056153.2:p.Tyr591Ter |
| 10062-3076 | Missense_mutation | PHF6 | X | NP_115834.1:p.Ile314Thr |
| 10062-3056 | Missense_mutation | EZH2 | 7 | NP_004447.2:p.Phe729Ser |
| 10062-3056 | Missense_mutation | JAK2 | 9 | NP_004963.1:p.Val617Phe |
| 10035-3142 | Missense_mutation | MPL | 1 | NP_005364.1:p.Trp515Leu |
| 10035-3109 | Missense_mutation | JAK2 | 9 | NP_004963.1:p.Val617Phe |
| 10058-3009 | Missense_mutation | JAK2 | 9 | NP_004963.1:p.Tyr613His |
| 10062-3073 | Missense_mutation | JAK2 | 9 | NP_004963.1:p.Val617Phe |
| 10062-3073 | Missense_mutation | IDH2 | 15 | NP_002159.2:p.Arg140Gln |
| 10013-3064 | Missense_mutation | SF3B1 | 2 | NP_036565.2:p.Lys666Thr |
| 10013-3064 | Missense_mutation | TET2 | 4 | NP_001120680.1:p.His1416Asp |
| 10013-3064 | Missense_mutation | JAK2 | 9 | NP_004963.1:p.Val617Phe |
| 10013-3064 | Missense_mutation | PHF6 | X | NP_115834.1:p.Ile314Thr |
| 10046-3057 | Missense_mutation | SETBP1 | 18 | NP_056374.2:p.Asp868Asn |
| 10046-3057 | Missense_mutation | U2AF1 | 21 | NP_001020374.1:p.Gln157Pro |
| 10046-3071 | Missense_mutation | JAK2 | 9 | NP_004963.1:p.Val617Phe |
| 10046-3071 | Missense_mutation | KRAS | 12 | NP_203524.1:p.Gln61Pro |
| 10046-3071 | Missense_mutation | U2AF1 | 21 | NP_001020374.1:p.Ser34Phe |
| 10046-3071 | Missense_mutation | U2AF1 | 21 | NP_001020374.1:p.Gln157Pro |
| 10046-3011 | Missense_mutation | JAK2 | 9 | NP_004963.1:p.Val617Phe |
| 10046-3011 | Nonsense_mutation | ASXL1 | 20 | NP_056153.2:p.Gln1350Ter |
| 10046-3011 | Missense_mutation | U2AF1 | 21 | NP_001020374.1:p.Ser34Phe |
| 10062-3134 | Frame_Shift_Del | TET2 | 4 | NP_001120680.1:p.Asn598LysfsTer2 |
| 10062-3134 | Missense_mutation | JAK2 | 9 | NP_004963.1:p.Val617Phe |
| 10062-3123 | Frame_Shift_Ins | TET2 | 4 | NP_001120680.1:p.Ser1392GlnfsTer9 |
| 10062-3123 | Frame_Shift_Ins | CALR | 19 | NP_004334.1:p.Lys385AsnfsTer47 |
| 10059-3124 | Frame_Shift_Del | TET2 | 4 | NP_001120680.1:p.Glu1250ArgfsTer3 |
| 10059-3124 | Missense_mutation | TET2 | 4 | NP_001120680.1:p.Ile1873Thr |
| 10059-3124 | Missense_mutation | JAK2 | 9 | NP_004963.1:p.Val617Phe |
| 10059-3124 | Missense_mutation | U2AF1 | 21 | NP_001020374.1:p.Ser34Phe |
| 10059-3145 | Missense_mutation | JAK2 | 9 | NP_004963.1:p.Val617Phe |
| 10059-3145 | Missense_mutation | TP53 | 17 | NP_000537.3:p.Cys176Phe |
| 34003-3077 | Missense_mutation | JAK2 | 9 | NP_004963.1:p.Val617Phe |
| 34003-3077 | Frame_Shift_Del | ASXL1 | 20 | NP_056153.2:p.Ala735LeufsTer9 |
| 34003-3077 | Missense_mutation | U2AF1 | 21 | NP_001020374.1:p.Ser34Tyr |
| 34003-3077 | Missense_mutation | IDH2 | 15 | NP_002159.2:p.Arg140Gln |
| 34001-3060 | Frame_Shift_Del | CALR | 19 | NP_004334.1:p.Leu367ThrfsTer46 |
| 39001-3087 | Missense_mutation | JAK2 | 9 | NP_004963.1:p.Val617Phe |
| 39001-3174 | Missense_mutation | JAK2 | 9 | NP_004963.1:p.Val617Phe |
| 39001-3174 | Nonsense_mutation | ASXL1 | 20 | NP_056153.2:p.Gln829Ter |
| 39001-3067 | Missense_mutation | JAK2 | 9 | NP_004963.1:p.Val617Phe |
| 44007-3200 | Frame_Shift_Ins | ASXL1 | 20 | NP_056153.2:p.Gly646TrpfsTer12 |
| 44007-3200 | Frame_Shift_Del | CALR | 19 | NP_004334.1:p.Leu367ThrfsTer46 |
| 44007-3192 | Missense_mutation | MPL | 1 | NP_005364.1:p.Trp515Lys |
| 44007-3192 | Frame_Shift_Ins | ASXL1 | 20 | NP_056153.2:p.Gly646TrpfsTer12 |
| 44001-3189 | Missense_mutation | JAK2 | 9 | NP_004963.1:p.Val617Phe |
| 44001-3189 | In_Frame_Del | SRSF2 | 17 | NP_001182356.1:p.Pro95_Arg102del |
| 44001-3189 | Missense_mutation | SETBP1 | 18 | NP_056374.2:p.Gly870Ser |
| 33010-3149 | Missense_mutation | JAK2 | 9 | NP_004963.1:p.Val617Phe |
| 33010-3181 | Missense_mutation | MPL | 1 | NP_005364.1:p.Trp515Leu |
| 33010-3181 | Missense_mutation | NRAS | 1 | NP_002515.1:p.Gly12Val |
| 33010-3181 | Missense_mutation | U2AF1 | 21 | NP_001020374.1:p.Gln157Pro |
| 33010-3181 | Frame_Shift_Del | ASXL1 | 20 |  |
| 44005-3183 | Frame_Shift_Del | CALR | 19 | NP_004334.1:p.Leu367ThrfsTer46 |
| 44009-3178 | Missense_mutation | JAK2 | 9 | NP_004963.1:p.Val617Phe |
| 44009-3178 | Missense_mutation | SF3B1 | 2 | NP_036565.2:p.Thr717Ala |
| 44009-3178 | Missense_mutation | TET2 | 4 | NP_001120680.1:p.Glu1207Gly |
| 44009-3178 | Splice_site | TET2 | 4 |  |
| 44009-3178 | Missense_mutation | TP53 | 17 | NP_000537.3:p.Arg175His |
| 44009-3198 | Missense_mutation | SF3B1 | 2 | NP_036565.2:p.Lys666Asn |
| 44009-3198 | In_Frame_Del | PHF6 | X | NP_115834.1:p.Glu338del |
| 44009-3198 | Frame_Shift_Ins | CALR | 19 | NP_004334.1:p.Lys385AsnfsTer47 |
| 33001-3177 | Missense_mutation | JAK2 | 9 | NP_004963.1:p.Val617Phe |
| 33001-3179 | Missense_mutation | JAK2 | 9 | NP_004963.1:p.Val617Phe |
| 39004-3088 | Missense_mutation | MPL | 1 | NP_005364.1:p.Trp515Leu |
| 39004-3088 | Missense_mutation | CBL | 11 | NP_005179.2:p.Cys416Ser |
| 39004-3088 | Missense_mutation | KRAS | 12 | NP_203524.1:p.Gln61Arg |
| 39004-3088 | Frame_Shift_Ins | ASXL1 | 20 | NP_056153.2:p.Gly646TrpfsTer12 |
| 39004-3088 | Missense_mutation | RUNX1 | 21 | NP_001745.2:p.Ala142Pro |
| 39004-3088 | Missense_mutation | U2AF1 | 21 | NP_001020374.1:p.Gln157Pro |
| 39004-3088 | Missense_mutation | NRAS | 1 | NP_002515.1:p.Gly12Asp |
| 39004-3088 | Nonsense_mutation | TET2 | 4 | NP_001120680.1:p.Gln916Ter |
| 39004-3088 | Missense_mutation | KRAS | 12 | NP_203524.1:p.Ala18Asp |
| 33001-3180 | Missense_mutation | JAK2 | 9 | NP_004963.1:p.Val617Phe |
| 33001-3180 | Missense_mutation | IDH2 | 15 | NP_002159.2:p.Arg140Gln |
| 33001-3180 | Missense_mutation | SRSF2 | 17 | NP_001182356.1:p.Pro95Leu |
| 39001-3196 | Missense_mutation | NRAS | 1 | NP_002515.1:p.Gly12Arg |
| 39001-3196 | Frame_Shift_Ins | TET2 | 4 | NP_001120680.1:p.Thr221AsnfsTer4 |
| 39001-3196 | Missense_mutation | JAK2 | 9 | NP_004963.1:p.Val617Phe |
| 39001-3196 | Frame_Shift_Ins | ASXL1 | 20 | NP_056153.2:p.Gly646TrpfsTer12 |
| 39001-3190 | Missense_mutation | MPL | 1 | NP_005364.1:p.Trp515Leu |
| 39001-3190 | Frame_Shift_Del | ASXL1 | 20 | NP_056153.2:p.Leu1213IlefsTer3 |
| 39001-3190 | Missense_mutation | EZH2 | 7 | NP_004447.2:p.Gly660Glu |
| 10068-3184 | Missense_mutation | SF3B1 | 2 | NP_036565.2:p.Ala744Pro |
| 10068-3184 | Missense_mutation | JAK2 | 9 | NP_004963.1:p.Val617Phe |
| 10068-3184 | Nonsense_mutation | TET2 | 4 | NP_001120680.1:p.Ser890Ter |
| 10062-3091 | Missense_mutation | JAK2 | 9 | NP_004963.1:p.Val617Phe |
| 10062-3091 | Missense_mutation | TP53 | 17 | NP_000537.3:p.Cys176Phe |
| 10035-3201 | Nonsense_mutation | TET2 | 4 | NP_001120680.1:p.Gln1547Ter |
| 10035-3201 | Missense_mutation | JAK2 | 9 | NP_004963.1:p.Val617Phe |
| 10014-3203 | Missense_mutation | SF3B1 | 2 | NP_036565.2:p.Arg625Leu |
| 10014-3203 | Missense_mutation | JAK2 | 9 | NP_004963.1:p.Val617Phe |
| 10014-3203 | Missense_mutation | PHF6 | X | NP_115834.1:p.Met278Lys |
| 10039-3187 | Frame_Shift_Del | TET2 | 4 | NP_001120680.1:p.Pro419LeufsTer8 |
| 10039-3187 | Frame_Shift_Del | ASXL1 | 20 | NP_056153.2:p.Glu635ArgfsTer15 |
| 10039-3187 | Frame_Shift_Del | CALR | 19 | NP_004334.1:p.Leu367ThrfsTer46 |
| 10065-3147 | Missense_mutation | TET2 | 4 | NP_001120680.1:p.Cys1135Tyr |
| 10065-3147 | Missense_mutation | JAK2 | 9 | NP_004963.1:p.Val617Phe |
| 10065-3147 | Frame_Shift_Del | PHF6 | X | NP_115834.1:p.Gln121ArgfsTer16 |
| 10065-3147 | Missense_mutation | NRAS | 1 | NP_002515.1:p.Gly13Arg |
| 10046-3199 | Missense_mutation | SF3B1 | 2 | NP_036565.2:p.Lys666Asn |
| 10046-3199 | Missense_mutation | JAK2 | 9 | NP_004963.1:p.Val617Phe |
| 10046-3159 | Missense_mutation | SF3B1 | 2 | NP_036565.2:p.His662Asp |
| 10046-3159 | Missense_mutation | JAK2 | 9 | NP_004963.1:p.Val617Phe |
| 10046-3159 | Missense_mutation | IDH2 | 15 | NP_002159.2:p.Arg140Gln |
| 10046-3156 | Frame_Shift_Ins | TET2 | 4 | NP_001120680.1:p.Leu200HisfsTer5 |
| 10046-3156 | Missense_mutation | JAK2 | 9 | NP_004963.1:p.Val617Phe |
| 10049-3128 | Missense_mutation | JAK2 | 9 | NP_004963.1:p.Val617Phe |
| 10049-3128 | Missense_mutation | U2AF1 | 21 | NP_001020374.1:p.Ser34Phe |
| 10049-3128 | Frame_Shift_Del | ASXL1 | 20 | NP_056153.2:p.Leu721CysfsTer4 |
| 10002-3165 | Nonsense_mutation | TET2 | 4 | NP_001120680.1:p.Gln1274Ter |
| 10002-3165 | Missense_mutation | JAK2 | 9 | NP_004963.1:p.Val617Phe |
| 10002-3165 | Splice_site | TET2 | 4 |  |
| 10002-3165 | Frame_Shift_Del | TET2 | 4 | NP_001120680.1:p.Asn1346IlefsTer17 |
| 10048-3188 | Missense_mutation | JAK2 | 9 | NP_004963.1:p.Val617Phe |
| 10048-3188 | Missense_mutation | IDH2 | 15 | NP_002159.2:p.Arg140Gln |
| 10048-3188 | Missense_mutation | TP53 | 17 | NP_000537.3:p.Gly266Glu |
| 10048-3188 | Missense_mutation | TP53 | 17 | NP_000537.3:p.Tyr163Asn |
| 10048-3188 | Frame_Shift_Ins | ASXL1 | 20 | NP_056153.2:p.Gly646TrpfsTer12 |
| 10048-3188 | Missense_mutation | U2AF1 | 21 | NP_001020374.1:p.Gln157Pro |
| 10048-3090 | Missense_mutation | JAK2 | 9 | NP_004963.1:p.Val617Phe |
| 10048-3090 | Missense_mutation | TET2 | 4 | NP_001120680.1:p.Thr1884Ala |
| 10046-3160 | Missense_mutation | SF3B1 | 2 | NP_036565.2:p.Lys700Glu |
| 10046-3160 | Frame_Shift_Ins | CALR | 19 | NP_004334.1:p.Lys385AsnfsTer47 |
| 10046-3160 | Missense_mutation | TP53 | 17 | NP_000537.3:p.Arg248Trp |
| 10060-3168 | Nonsense_mutation | TET2 | 4 | NP_001120680.1:p.Gln1652Ter |
| 10060-3168 | Frame_Shift_Ins | EZH2 | 7 | NP_004447.2:p.Asn263GlnfsTer8 |
| 10060-3168 | Frame_Shift_Ins | ASXL1 | 20 | NP_056153.2:p.Gly646TrpfsTer12 |
| 10060-3168 | Frame_Shift_Del | CALR | 19 | NP_004334.1:p.Leu367ThrfsTer46 |
| 10060-3168 | Missense_mutation | IDH1 | 2 | NP_005887.2:p.Arg132Cys |
| 10060-3168 | Missense_mutation | CBL | 11 | NP_005179.2:p.Cys396Ser |
| 10060-3168 | Missense_mutation | CBL | 11 | NP_005179.2:p.Cys419Tyr |
| 10064-3193 | Missense_mutation | JAK2 | 9 | NP_004963.1:p.Val617Phe |
| 10064-3193 | Frame_Shift_Ins | ASXL1 | 20 | NP_056153.2:p.Gly646TrpfsTer12 |
| 10064-3193 | Frame_Shift_Ins | PHF6 | X | p.Ter366Serext*21 |
| 10059-3157 | Missense_mutation | JAK2 | 9 | NP_004963.1:p.Val617Phe |
| 10059-3157 | Missense_mutation | NRAS | 1 | NP_002515.1:p.Gly12Asp |
| 10059-3157 | Missense_mutation | RUNX1 | 21 | NP_001745.2:p.Asp160Tyr |
| 10059-3166 | Missense_mutation | SF3B1 | 2 | NP_036565.2:p.Lys666Arg |
| 10059-3166 | Missense_mutation | JAK2 | 9 | NP_004963.1:p.Val617Phe |
| 10073-3194 | Missense_mutation | SF3B1 | 2 | NP_036565.2:p.Lys666Thr |
| 10073-3194 | Frame_Shift_Del | ASXL1 | 20 | NP_056153.2:p.Gly949ValfsTer2 |
| 10073-3194 | Frame_Shift_Del | CALR | 19 | NP_004334.1:p.Leu367ThrfsTer46 |
| 10012-3171 | Missense_mutation | JAK2 | 9 | NP_004963.1:p.Val617Phe |
| 10022-3234 | Missense_mutation | JAK2 | 9 | NP_004963.1:p.Val617Phe |
| 10035-3206 | Nonsense_mutation | TET2 | 4 | NP_001120680.1:p.Leu1816Ter |
| 10035-3206 | Missense_mutation | JAK2 | 9 | NP_004963.1:p.Val617Phe |
| 10022-3221 | Missense_mutation | KRAS | 12 | NP_203524.1:p.Gly12Ser |
| 10022-3221 | Missense_mutation | KRAS | 12 | NP_203524.1:p.Gly12Arg |
| 10022-3221 | Frame_Shift_Del | CALR | 19 | NP_004334.1:p.Leu367ThrfsTer46 |
| 10039-3244 | Missense_mutation | JAK2 | 9 | NP_004963.1:p.Val617Phe |
| 10039-3244 | Frame_Shift_Ins | ASXL1 | 20 | NP_056153.2:p.Gly646TrpfsTer12 |
| 10039-3244 | Missense_mutation | U2AF1 | 21 | NP_001020374.1:p.Gln157Pro |
| 10046-3213 | Missense_mutation | EZH2 | 7 | NP_004447.2:p.His158Pro |
| 10046-3213 | Missense_mutation | JAK2 | 9 | NP_004963.1:p.Val617Phe |
| 10046-3213 | Frame_Shift_Ins | ASXL1 | 20 | NP_056153.2:p.Gly646TrpfsTer12 |
| 10062-3231 | Missense_mutation | DNMT3A | 2 | NP_072046.2:p.Arg882Ser |
| 10062-3231 | Missense_mutation | SF3B1 | 2 | NP_036565.2:p.Lys700Glu |
| 10062-3231 | Missense_mutation | JAK2 | 9 | NP_004963.1:p.Val617Phe |
| 10062-3231 | Missense_mutation | TET2 | 4 | NP_001120680.1:p.His269Arg |
| 10062-3231 | Frame_Shift_Del | TET2 | 4 | NP_001120680.1:p.Gln557SerfsTer4 |
| 10062-3231 | Frame_Shift_Ins | TET2 | 4 | NP_001120680.1:p.Cys1358LeufsTer43 |
| 10062-3225 | Missense_mutation | MPL | 1 | NP_005364.1:p.Trp515Lys |
| 10066-3237 | Missense_mutation | MPL | 1 | NP_005364.1:p.Trp515Leu |
| 10062-3210 | Missense_mutation | EZH2 | 7 | NP_004447.2:p.Arg690His |
| 10062-3210 | Missense_mutation | EZH2 | 7 | NP_004447.2:p.Asn130Lys |
| 10062-3210 | Missense_mutation | KRAS | 12 | NP_203524.1:p.Gln61Pro |
| 10062-3210 | Frame_Shift_Del | ASXL1 | 20 | NP_056153.2:p.Glu635ArgfsTer15 |
| 10025-3218 | Missense_mutation | JAK2 | 9 | NP_004963.1:p.Val617Phe |
| 10025-3218 | Frame_Shift_Ins | ASXL1 | 20 | NP_056153.2:p.Gly646TrpfsTer12 |
| 10002-3209 | Missense_mutation | JAK2 | 9 | NP_004963.1:p.Val617Phe |
| 10014-3207 | Missense_mutation | JAK2 | 9 | NP_004963.1:p.Val617Phe |
| 10014-3207 | Frame_Shift_Ins | ASXL1 | 20 | NP_056153.2:p.Gly646TrpfsTer12 |
| 10014-3207 | Frame_Shift_Ins | ASXL1 | 20 | NP_056153.2:p.Ser846GlnfsTer5 |
| 10014-3207 | Missense_mutation | KRAS | 12 | NP_203524.1:p.Gly12Arg |
| 10014-3207 | Frame_Shift_Del | ASXL1 | 20 | NP_056153.2:p.Phe1373CysfsTer6 |
| 10035-3211 | Missense_mutation | NRAS | 1 | NP_002515.1:p.Gln61Arg |
| 10035-3211 | Frame_Shift_Del | CALR | 19 | NP_004334.1:p.Glu364ArgfsTer49 |
| 44001-3224 | Missense_mutation | SF3B1 | 2 | NP_036565.2:p.Lys666Asn |
| 44001-3224 | Missense_mutation | JAK2 | 9 | NP_004963.1:p.Val617Phe |
| 44001-3224 | Nonsense_mutation | ASXL1 | 20 | NP_056153.2:p.Arg965Ter |
| 44001-3228 | Frame_Shift_Del | TET2 | 4 | NP_001120680.1:p.Leu1231TrpfsTer22 |
| 44001-3228 | Missense_mutation | JAK2 | 9 | NP_004963.1:p.Val617Phe |
| 44001-3228 | Nonsense_mutation | ASXL1 | 20 | NP_056153.2:p.Arg1415Ter |
| 44001-3228 | Splice_site | ZRSR2 | X | NP_005080.1 |
| 39001-3222 | Missense_mutation | JAK2 | 9 | NP_004963.1:p.Val617Phe |
| 44009-3215 | Missense_mutation | JAK2 | 9 | NP_004963.1:p.Val617Phe |
| 34005-3235 | Missense_mutation | JAK2 | 9 | NP_004963.1:p.Val617Phe |
| 44001-3227 | Nonsense_mutation | TET2 | 4 | NP_001120680.1:p.Gln1537Ter |
| 44001-3227 | Nonsense_mutation | TET2 | 4 | NP_001120680.1:p.Cys1875Ter |
| 44001-3227 | Frame_Shift_Del | CALR | 19 | NP_004334.1:p.Leu367ThrfsTer46 |
| 34001-3230 | Missense_mutation | JAK2 | 9 | NP_004963.1:p.Val617Phe |
| 34001-3230 | Frame_Shift_Ins | ASXL1 | 20 | NP_056153.2:p.Gly646TrpfsTer12 |
| 34001-3230 | Nonsense_mutation | ASXL1 | 20 | NP_056153.2:p.Arg965Ter |
| 44006-3236 | Missense_mutation | JAK2 | 9 | NP_004963.1:p.Val617Phe |
| 44006-3236 | Missense_mutation | KRAS | 12 | NP_203524.1:p.Gln61Pro |
| 10063-3243 | Missense_mutation | JAK2 | 9 | NP_004963.1:p.Val617Phe |
| 33002-3122 | Missense_mutation | SF3B1 | 2 | NP_036565.2:p.Lys700Glu |
| 33002-3122 | Missense_mutation | TP53 | 17 | NP_000537.3:p.Tyr163Cys |
| 33002-3122 | Frame_Shift_Del | CALR | 19 | NP_004334.1:p.Leu367ThrfsTer46 |
| 46001-3120 | Missense_mutation | NRAS | 1 | NP_002515.1:p.Gly12Cys |
| 46001-3120 | Missense_mutation | JAK2 | 9 | NP_004963.1:p.Val617Phe |
| 10022-3175 | Nonsense_mutation | TET2 | 4 | NP_001120680.1:p.Gly1735Ter |
| 10022-3175 | Missense_mutation | JAK2 | 9 | NP_004963.1:p.Val617Phe |
| 10015-3117 | Missense_mutation | JAK2 | 9 | NP_004963.1:p.Val617Phe |
| 10015-3117 | Missense_mutation | KRAS | 12 | NP_203524.1:p.Gly12Val |
| 10015-3117 | Nonsense_mutation | ASXL1 | 20 | NP_056153.2:p.Trp1037Ter |
| 44014-3208 | Missense_mutation | JAK2 | 9 | NP_004963.1:p.Val617Phe |
| 10073-3212 | Missense_mutation | JAK2 | 9 | NP_004963.1:p.Val617Phe |
| 10073-3212 | Frame_Shift_Del | ASXL1 | 20 | NP_056153.2:p.Glu635ArgfsTer15 |
| 10073-3212 | Nonsense_mutation | ASXL1 | 20 | NP_056153.2:p.Arg693Ter |
| 10006-3219 | Missense_mutation | JAK2 | 9 | NP_004963.1:p.Val617Phe |
| 10006-3216 | Missense_mutation | JAK2 | 9 | NP_004963.1:p.Val617Phe |
| 10006-3216 | Missense_mutation | KRAS | 12 | NP_203524.1:p.Tyr64Asp |
| 10006-3216 | Nonsense_mutation | ASXL1 | 20 | NP_056153.2:p.Gln858Ter |
| 10006-3216 | Splice_site | ZRSR2 | X |  |

**Table S1. Pathogenic variants in the PAC203 cohort.** Chr=chromosome.
