# Supplementary material for: RAS-pathway mutations are common in patients with ruxolitinib refractory/intolerant myelofibrosis: molecular analysis of the PAC203 cohort
Source: Leukemia. 2023 Oct 20;37(12):2497–501. doi: 10.1038/s41375-023-02027-3 (PMC10681886; doi:10.1038/s41375-023-02027-3)
Supplement: Supplementary file 3 — Supplemental Table 2A [file 41375_2023_2027_MOESM3_ESM.docx]

| **Baseline Characteristics n (%)** | **SF-mutated** | **SF-WT** |  |
| --- | --- | --- | --- |
|  | **N=38** | **N=72** | ***P* value** |
| **Age in years, median (range)** | 66.5 (46-87) | 68.5 (37-85) | 0.722 |
| **Male gender** | 27 (71.1) | 37 (51.4) | 0.047 |
| **MF diagnosis** |  | | |
| Primary MF | 29 (76.3) | 33 (45.8) | 0.001 |
| PPV MF | 3 (7.9) | 29 (40.3) |  |
| PET MF | 6 (15.8) | 10 (13.9) |  |
| **Prior ruxolitinib failure** | 25 (65.8) | 55 (76.4) | 0.189 |
| **Prior ruxolitinib intolerance** | 30 (78.9) | 47 (65.3) | 0.137 |
| **Ruxolitinib exposure in months, median (range)** | 12.4 (2.1-63.1) | 24 (1.7-131.4) | 0.058 |
| **Hemoglobin <10g/dL** | 31 (81.6) | 40 (55.6) | 0.008 |
| **Platelet count <50, x 10^9^/L** | 16 (42.1) | 26 (36.1) | 0.575 |
| **Platelet count x 10^9^/L, median (range)** | 61.5 (15-359) | 63 (13-910) | 0.597 |
| **White blood cells, ×10^9^/L, median (range)** | 6.8 (2.3, 61.5) | 7 (1.1-107.7) | 0.191 |
| **Peripheral blast %, median (range)** | 2 (0-17) | 2 (0-9) | 0.393 |
| **RBC transfusion-dependent** | 16 (42.1) | 16 (22.2) | 0.012 |
| **Platelet transfusion-dependent** | 5 (13.5) | 4 (5.6) | 0.153 |
| **Spleen Volume (cm^3^) by MRI/CT, median (range)** | 2421 (458-4994) | 2434 (262, 5521) | 0.8 |
| **Driver mutation status** |  | | |
| *JAK2* V617F | 30 (78.9) | 55 (76.4) | 0.971 |
| *CALR* | 4 (10.5) | 10 (13.9) |  |
| *MPL* | 3 (7.9) | 6 (8.3) |  |
| Triple negative | 1 (2.6) | 1 (1.4) |  |
| ***JAK2* V617F allele burden ≥50%** | 17 (45.9) | 46 (64.8) | 0.059 |

**Table S2A. Splicing factor (SF) mutated patient baseline clinical and mutation characteristics.** WT=wild-type; MF=myelofibrosis; PPV=post polycythemia, PET=post essential thrombocythemia; RBC=red blood cell.
