# Supplementary material for: RAS-pathway mutations are common in patients with ruxolitinib refractory/intolerant myelofibrosis: molecular analysis of the PAC203 cohort
Source: Leukemia. 2023 Oct 20;37(12):2497–501. doi: 10.1038/s41375-023-02027-3 (PMC10681886; doi:10.1038/s41375-023-02027-3)
Supplement: Supplementary file 4 — Supplemental Table 2B [file 41375_2023_2027_MOESM4_ESM.docx]

| **Baseline Characteristics n (%)** | ***HMR-*mutated** | ***HMR-*WT** |  |
| --- | --- | --- | --- |
|  | **N=48** | **N=62** | ***P* value** |
| **Age in years, median (range)** | 68.5 (51-85) | 66.5 (37-87) | 0.308 |
| **Male gender** | 32 (66.7) | 32 (51.6) | 0.112 |
| **MF diagnosis** |  | | |
| Primary MF | 26 (54.2) | 36 (58.1) | 0.542 |
| PPV MF | 13 (27.1) | 19 (30.6) |  |
| PET MF | 9 (18.8) | 7 (11.3) |  |
| **Prior ruxolitinib failure** | 36 (75) | 44 (71) | 0.737 |
| **Prior ruxolitinib intolerance** | 33 (68.8) | 3 (68.9) | 0.801 |
| **Ruxolitinib exposure in months, median (range)** | 17.5 (2.1-84.2) | 20.1 (1.7-131.4) | 0.729 |
| **Hemoglobin <10g/dL** | 31 (66) | 40 (64.5) | 0.876 |
| **Platelet count <50, x 10^9^/L** | 14 (29.2) | 28 (45.9) | 0.075 |
| **Platelet count x 10^9^/L, median (range)** | 89 (15-402) | 52 (13-910) | 0.079 |
| **White blood cells, ×10^9^/L, median (range)** | 6.8 (1.1-107.7) | 7.1 (1.2-103.4) | 0.615 |
| **Peripheral blast %, median (range)** | 2 (0-9) | 2 (0-17) | 0.231 |
| **RBC transfusion-dependent** | 16 (34.8) | 16 (25.8) | 0.419 |
| **Platelet transfusion-dependent** | 4 (8.5) | 5 (8.1) | 0.933 |
| **Spleen Volume (cm^3^) by MRI/CT, median (range)** | 2240 (458-5520) | 2454 (262-4951) | 0.635 |
| **Driver mutation status** |  | | |
| *JAK2* V617F | 36 (75) | 49 (79) | 0.319 |
| *CALR* | 5 (10.4) | 9 (14.5) |  |
| *MPL* | 5 (10.4) | 4 (6.5) |  |
| Triple negative | 2 (4.2) | 0 (0) |  |
| ***JAK2* V617F allele burden ≥50%** | 28 (60.9) | 35 (56.5) | 0.64 |

**Table S2B. High molecular risk (HMR)-mutated patient baseline clinical and mutation characteristics.** WT=wild-type; MF=myelofibrosis; PPV=post polycythemia, PET=post essential thrombocythemia; RBC=red blood cell.
